# Whole pelvis vs. hemi pelvis elective nodal radiotherapy in patients with PSMA-positive nodal recurrence after radical prostatectomy - a retrospective multi-institutional propensity score analysis

**DOI:** 10.1007/s00259-024-06802-x

**Published:** 2024-06-28

**Authors:** Christian Trapp, Daniel M. Aebersold, Claus Belka, Jozefina Casuscelli, Louise Emmett, Chukwuka Eze, Stefano Fanti, Andrea Farolfi, Wolfgang Fendler, Anca-Ligia Grosu, Matthias Guckenberger, George Hruby, Simon Kirste, Stefan A. Koerber, Stephanie Kroeze, Jan C. Peeken, Paul Rogowski, Sophia Scharl, Mohamed Shelan, Simon K. B. Spohn, Iosif Strouthos, Lena Unterrainer, Marco Vogel, Thomas Wiegel, Constantinos Zamboglou, Nina-Sophie Schmidt-Hegemann

**Affiliations:** 1 Department of Radiation Oncology, University Hospital, LMU Munich, Marchioninistr. 15, 81377 Munich, Germany; 2 Department of Radiation Oncology, Inselspital, Bern University Hospital, University of Bern, Bern, Switzerland; 3 Deutsches Konsortium für Translationale Krebsforschung (DKTK), Partner Site Munich, Munich, Germany; 4 Department of Urology, University Hospital, LMU Munich, Munich, Germany; 5 Department of Theranostics and Nuclear Medicine, St. Vincent’s Hospital, Sydney, Australia; 6 St. Vincent’s Clinical School, University of New South Wales, Sydney, Australia; 7 Nuclear Medicine, IRCCS Azienda Ospedaliero-Universitaria di Bologna, Bologna, Italy; 8 Department of Nuclear Medicine, University Hospital,University of Essen, Essen, Germany; 9 Department of Radiation Oncology, Medical Center , University of Freiburg, Freiburg, Germany; 10 Deutsches Konsortium für Translationale Krebsforschung (DKTK), Partner Site Freiburg, Freiburg, Germany; 11 Department of Radiation Oncology, University Hospital, University of Zurich, Zurich, Switzerland; 12 Department of Radiation Oncology, Royal North Shore Hospital, University of Sydney, Sydney, Australia; 13 Department of Radiation Oncology, Barmherzige Brüder Hospital Regensburg, Regensburg, Germany; 14 Department of Radiation Oncology, Heidelberg University Hospital, Heidelberg, Germany; 15 Department of Radiation Oncology, Klinikum rechts der Isar, Technical University of Munich (TUM), Munich, Germany; 16 Department of Radiation Oncology, University of Ulm, Ulm, Germany; 17 Berta-Ottenstein-Programm, Medical Faculty, University of Freiburg, Freiburg, Germany; 18 Department of Radiation Oncology, German Oncology Center, European University Cyprus, Nicosia, Cyprus; 19 Department of Nuclear Medicine, University Hospital, LMU Munich, Munich, Germany; 20 Ahmanson Translational Theranostics Division, Department of Molecular and Medical Pharmacology, David Geffen School of Medicine, UCLA, Los Angeles, USA

**Keywords:** Prostate cancer, Nodal recurrence, PSMA PET/CT, Radiotherapy, Hemi pelvis, Whole pelvis

## Abstract

**Purpose:**

Despite growing evidence for bilateral pelvic radiotherapy (whole pelvis RT, WPRT) there is almost no data on unilateral RT (hemi pelvis RT, HPRT) in patients with nodal recurrent prostate cancer after prostatectomy. Nevertheless, in clinical practice HPRT is sometimes used with the intention to reduce side effects compared to WPRT. Prostate-specific membrane antigen positron emission tomography / computed tomography (PSMA-PET/CT) is currently the best imaging modality in this clinical situation. This analysis compares PSMA-PET/CT based WPRT and HPRT.

**Methods:**

A propensity score matching was performed in a multi-institutional retrospective dataset of 273 patients treated with pelvic RT due to nodal recurrence (214 WPRT, 59 HPRT). In total, 102 patients (51 in each group) were included in the final analysis. Biochemical recurrence-free survival (BRFS) defined as prostate specific antigen (PSA) < post-RT nadir + 0.2ng/ml, metastasis-free survival (MFS) and nodal recurrence-free survival (NRFS) were calculated using the Kaplan-Meier method and compared using the log rank test.

**Results:**

Median follow-up was 29 months. After propensity matching, both groups were mostly well balanced. However, in the WPRT group there were still significantly more patients with additional local recurrences and biochemical persistence after prostatectomy. There were no significant differences between both groups in BRFS (*p* = .97), MFS (*p* = .43) and NRFS (*p* = .43). After two years, BRFS, MFS and NRFS were 61%, 86% and 88% in the WPRT group and 57%, 90% and 82% in the HPRT group, respectively. Application of a boost to lymph node metastases, a higher RT dose to the lymphatic pathways (> 50 Gy EQD2_α/β=1.5 Gy_) and concomitant androgen deprivation therapy (ADT) were significantly associated with longer BRFS in uni- and multivariate analysis.

**Conclusions:**

Overall, this analysis presents the outcome of HPRT in nodal recurrent prostate cancer patients and shows that it can result in a similar oncologic outcome compared to WPRT. Nevertheless, patients in the WPRT may have been at a higher risk for progression due to some persistent imbalances between the groups. Therefore, further research should prospectively evaluate which subgroups of patients are suitable for HPRT and if HPRT leads to a clinically significant reduction in toxicity.

## Background

After radical prostatectomy, approximately one third of prostate cancer (PC) patients have a biochemical persistence or develop a biochemical recurrence [1, 2]. Nowadays, in this situation many patients are staged with prostate-specific membrane antigen positron emission tomography / computed tomography (PSMA-PET/CT), which often reveals lymph node metastases (LNM) [3]. The optimal therapeutic strategies are still subject to ongoing discussion, reaching from androgen deprivation therapy (ADT), salvage lymph node dissection (sLND) to radiotherapeutic approaches. The latter include stereotactic body radiotherapy (SBRT) or irradiation of the pelvic lymphatic pathways (whole pelvis RT, WPRT), possibly with a simultaneous integrated boost (SIB) to LNM. Regarding SBRT, there is evolving evidence from retrospective trials [4, 5] and prospective phase I and II trials in patients with oligometastatic prostate cancer (PC) which included many patients with LNM [6–10]. Regarding WPRT with SIB to LNM, there are some retrospective outcome analyses [11–14] and a prospective phase II trial reporting the outcome of WPRT + 6 months ADT [15]. Furthermore, a retrospective multi-institutional comparison between WPRT and SBRT showed a better metastasis-free survival (MFS) in patients treated with WPRT [16]. Randomized data can be awaited from the “Oligopelvis 2 GETUG P12” trial, which randomizes patients between intermittent ADT + WPRT vs. intermittent ADT alone (NCT03630666) and the “Peace V - Storm“ trial [17], which randomizes patients between 6 months ADT + metastasis directed therapy (MDT) (sLND or SBRT) vs. 6 months ADT + MDT + WPRT. Nevertheless, in the absence of definitive prospective evidence some institutions currently treat patients also with individualized concepts, e.g. an “involved field RT” [18, 19] or an unilateral RT of the pelvic lymphatic pathways on the side of the LNM (hemi pelvis RT, HPRT) with the intent to reduce the locoregional failure rate while keeping toxicity low. In this retrospective analysis we compare the results of PSMA-PET/CT guided HPRT with WPRT based on a large multi-institutional dataset allowing propensity score matching.

## Methods

### Patient population

This analysis is based on a retrospective dataset of eleven centers in five countries (6 German centers, 2 Swiss centers, 1 Australian center, 1 Cypriot center, 1 Italian center). The dataset includes 1222 PC patients who received PSMA-PET/CT guided salvage RT due to PSA recurrence or PSA persistence after prostatectomy. Details regarding PET/CT protocols, RT protocols and follow up concepts can be found in the supplements of an earlier publication [20]. In 407 of these 1222 patients PSMA-PET/CT detected LNM. After cleaning up the dataset by removing patients with insufficient or incomplete follow up and patients who did not receive a RT of the lymphatic pathways (e.g. patients who were treated by SBRT), the dataset contained 273 cases. 214 of these patients received WPRT while 59 patients received HPRT.

In a next step, propensity score matching was performed using IBM-SPSS^®^ version 29. Use of ADT (yes or no), prostate specific antigen (PSA) value before PSMA-PET/CT (≤ 0.2 ng/ml, 0.21–0.5 ng/ml, 0,51–1,0 ng/ml or > 1,0 ng/ml), age and ISUP-Score (≤ 3 or ≥ 4) were used as matching variables. A matching tolerance of 0,01 was chosen.

Propensity score matching resulted in a final dataset consisting of 102 patients (51 in each group). Regarding patients’ characteristics the groups of the final dataset still significantly differed in the proportion of patients with biochemical persistence after prostatectomy (43% in the WPRT vs. 16% in the HPRT group) and the presence of an additional local recurrence in PSMA-PET/CT (33% vs. 6%). Nevertheless, incorporating those parameters into the matching algorithm resulted in a very limited dataset with no precise matches so that we decided to proceed with the 102 patient - dataset.

### Ethics

Local ethics committees of participating centers approved this study (University of Freiburg, Freiburg, Germany; University of Munich, Munich, Germany; Technical University of Munich, Munich, Germany; University of Bologna, Bologna, Italy; University of Ulm, Ulm, Germany; Ethics Committee Zuerich, Zuerich, Switzerland; University of New South Wales, Sydney, Australia; University of Heidelberg, Heidelberg, Germany; Cyprus National Bioethics Committee, Nicosia, Cyprus; Cantonal Ethics Commission Bern, Bern, Switzerland). Written informed consent was waived due to the retrospective character of the study in accordance with all respective review boards.

### PET/CT

PET/CT acquisition and interpretation was performed in accordance with the local practice of all participating centers as shown in [20]. ^68^Ga-PSMA or ^18^F-PSMA were used.

### Statistics

Primary endpoint was biochemical recurrence-free survival (BRFS). Biochemical recurrence was defined as a rise of 0.2 ng/ml above the post-RT nadir. Secondary endpoints were MFS and nodal recurrence-free survival (NRFS). Metastases were defined as hematogenous metastases or supradiaphragmatic LNM. Nodal recurrence was defined as recurrence in a pelvic or paraaortic lymph node. Survival analyses were based on time from last day of RT to biochemical progression / diagnosis of a new metastasis, death or to the date of the last follow-up. Statistical analyses were conducted using IBM-SPSS® version 29. Survival analyses were calculated using the Kaplan-Meier method and compared using the log-rank test. Predictive factors for BRFS were evaluated by uni- and multivariate Cox regression analyses in the whole dataset (102 patients).

## Results

### Patients’ and treatment characteristics

After propensity score matching, the final dataset consisted of 102 patients (51 per group). Patients’ characteristics were mostly similar in both groups (see Table 1). Regarding T stage, N stage, R status and ISUP score there were no significant differences. The PSA value before PET/CT was similar in both groups, too. The only significant differences relate to the percentages of patients with PSA persistence after prostatectomy (43% in the WPRT group vs. 16% in the HPRT group) and with the finding of an additional local recurrence in the PSMA-PET/CT (33% in the WPRT vs. 6% in the HPRT group). Treatment characteristics were also mostly well balanced between both groups (see Table 1). The percentage of patients with additional RT of the prostate bed and the dose to the lymphatic pathways were similar as well as the percentage of patients with additional ADT and the duration of the ADT treatment. Regarding treatment characteristics, only the dose of the LNM boost differed significantly (59% vs. 0% received > 60 Gy equivalent dose in 2 Gy fractions (EQD2)_α/β=1.5 Gy_ in the WPRT and HPRT group, respectively). The median follow up was longer in the WPRT group (40 vs. 24 months).

**Table 1:** Patients’ and treatment characteristics

	WPRT	HPRT	
Age [years]	71 (53–78)	71 (51–87)	*p* = 0.95 ^1^
pT Stage			*p* = 0.34 ^2^
- pT2	21 (41%)	27 (53%)	
- pT3a	9 (18%)	11 (22%)	
- pT3b	16 (31%)	10 (20%)	
- pT4	1 (2%)	0	
- no data available	4 (8%)	3 (6%)	
pN Stage			*p* =0.28 ^3^
- pN0	34 (67%)	37 (73%)	
- pN1	11 (22%)	6 (12%)	
- no data available	6 (12%)	8 (16%)	
R Status			*p* = 0.88 ^3^
- R0	27 (53%)	37 (73%)	
- R1	15 (29%)	8 (16%)	
- no data available	9 (18%)	6 (12%)	
ISUP Score			*p*= 0.76 ^2^
- 1 or 2	15 (29%)	17 (33%)	
- 3	11 (22%)	14 (28%)	
- 4	15 (29%)	11 (22%)	
- 5	10 (20%)	9 (18%)	
PSA Persistence after RPE			***p*** **= 0.00**^**3**^
- persistence	22 (43%)	8 (16%)	
- recurrence	28 (55%)	41 (80%)	
- no data available	1 (2%)	2 (4%)	
PSA before PET/CT [ng / ml]			*p* = 0.84 ^2^
- ≤ 0.2	1 (2%)	1 (2%)	
- 0.21–0.5	11 (22%)	12 (24%)	
- 0.51–1.0	13 (26%)	9 (18%)	
- > 1.0	26 (51%)	29 (57%)	
Local Recurrence in PET/CT			***p*** **= 0.00**^**3**^
- no	34 (67%)	48 (94%)	
- yes	17 (33%)	3 (6%)	
RT of Prostate Bed			*p* = 0.29 ^3^
- yes	40 (78%)	40 (85%)	
- no	11 (22%)	7 (15%)	
Boost of LNM			*p* = 0.76 ^3^
- yes	46 (90%)	44 (86%)	
- no	5 (10%)	7 (14%)	
RT Dose to Lymphatic Pathways [Gy]			
			*p* = 0.59 ^3^
- EQD2_α/β=1.5 Gy_ ≤ 50 Gy	42 (82%)	44 (86%)	
- EQD2_α/β=1.5 Gy_ > 50 Gy	7 (14%)	7 (14%)	
- no data available	2 (4%)	0	
RT Dose to LNM [Gy]			***p*** **= 0 .00**^**2**^
- EQD2_α/β=1.5 Gy_ ≤ 50 Gy	1 (2%)	0	
- EQD2_α/β=1.5 Gy_ 50.1–60 Gy	12 (24%)	44 (86%)	
- EQD2_α/β=1.5 Gy_ > 60 Gy	30 (59%)	0	
- no data available	8 (16%)	7 (14%)	
ADT during RT			*p* =1.00 ^3^
- yes	35 (69%)	36 (71%)	
- no	16 (31%)	15 (29%)	
Duration of ADT			*p* = 0.38 ^2^
- ≤ 6 months	4 (8%)	2 (4%)	
- > 6 and ≤ 12 months	5 (10%)	8 (16%)	
- > 12 and ≤ 24 months	5 (10%)	2 (4%)	
- > 24 months	1 (2%)	3 (6%)	
- no data available	36 (71%)	36 (71%)	
Follow Up [months]	40 (14–84)	28 (12–64)	***p*** **= 0.00**^**1**^

^1^ = t-test, ^2^ = chi square test, ^3^ = Fisher’s exact test

### Biochemical recurrence-free survival

**Fig. 1:**
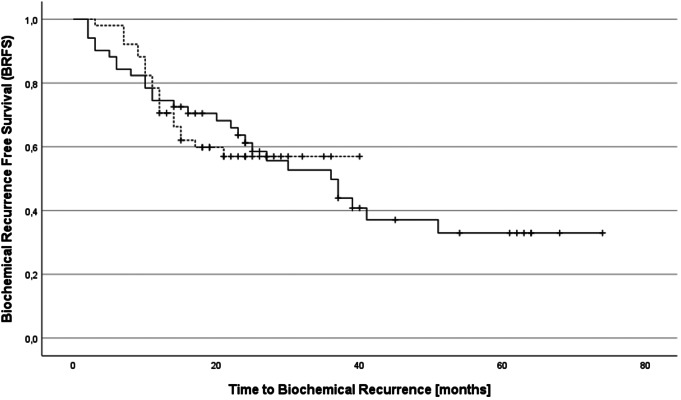
Biochemical recurrence-free survival (BRFS) in patients receiving whole pelvis RT (solid line) and hemi pelvis RT (broken line)

We did not observe significant differences in BRFS (*p* = .97). BRFS after two years was 61% in the WPRT group and 57% in the HPRT group. Median BRFS was 36 months in WPRT patients and was not reached in HPRT patients (see Fig. 1).

### Metastasis-free survival and nodal recurrence-free survival

**Fig. 2:**
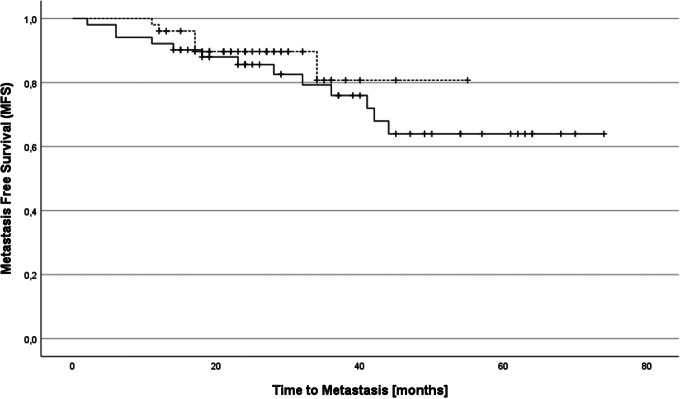
Metastasis-free survival (MFS) in patients receiving whole pelvis RT (solid line) and hemi pelvis RT (broken line)

**Fig. 3:**
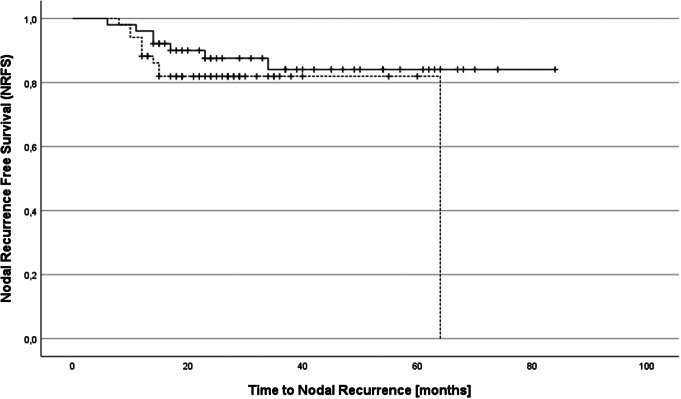
Nodal recurrence-free survival (NRFS) in patients receiving whole pelvis RT (solid line) and hemi pelvis RT (broken line)

MFS and NRFS were also similar in both groups (*p* = .43 for MFS, *p* = .23 for NRFS). After two years, MFS was 86% in the WPRT group vs. 90% in the HPRT group and NRFS was 88% in the WPRT group vs. 82% in the HPRT group, respectively. Regarding MFS and NRFS, medians were not reached in both groups (see Figs. 2 and 3).

### Univariate and multivariate analysis

Univariate analysis of the whole dataset (WPRT and HPRT) reveals that a RT dose to the lymphatic pathways of EQD2_α/β=1.5 Gy_ > 50 Gy, the application of a LNM boost, additional RT of the prostate bed and concomitant ADT were significant predictors of BRFS. In the multivariate Cox regression analysis of these factors all but additional RT to the prostate bed could be confirmed as significant predictors (see Table 2).

**Table 2:** Significant predictors of BRFS in the whole dataset (*n* = 102)

				Univariate Analysis		Multivariate Analysis	
**Patient Characteristics**		**n**	**Median BRFS [months]**	**HR (95% CI)**	*p*-value	**HR (95% CI)**	*p*-value
**pT Stage**				1.24 (0.69–2.21)	0.475		
pT2		48	39				
pT3 - pT4		47	27				
**pN Stage**				1.01 (0.47–2.17)	0.985		
pN0		71	36				
pN1		17	27				
**R Status**				0.70 (0.34–1.43)	0.327		
R0		64	30				
R1		23	39				
**ISUP Score**				0.71 (0.33–1.52)	0.380		
1–3		83	30				
4–5		19	37				
**PSA Persistence vs. PSA Recurrence**				1.07 (0.59–1.95)	0.828		
PSA persistence		30	37				
PSA recurrence		69	37				
**PSA before PET/CT**				1.27 (0.72–2.25)	0.412		
≤ 1 ng/ml		47	36				
> 1 ng/ml		55	37				
**Local Recurrence in PET/CT**				0.93 (0.46–1.86)	0.836		
no local recurrence		82	36				
local recurrence		20	37				
**RT of the Lymphatic Pathways**				1.01 (0.56–1.84)	0.967		
hemi pelvis RT		51	NR				
whole pelvis RT		51	36				
**RT Dose to the Lymphatic Pathways (EQD2**_**α/β=1.5 Gy**_ **)**				0.20 (0.05–0.81)	**0.024**	0.14 (0.03–0.57)	**0.007**
≤ 50 Gy		86	27				
> 50 Gy		14	NR				
**LNM Boost**				0.32 (0.14–0.75)	**0.008**	0.18 (0.06–0.52)	**0.002**
no LNM boost		12	8				
LNM boost		90	37				
**RT Dose to LNM (EQD2**_**α/β=1.5 Gy**_ **)**				0.60 (0.31–1.19)	0.143		
≤ 60 Gy		57	37				
> 60 Gy		30	37				
**RT of Prostate Bed**				0.49 (0.25–0.97)	**0.040**	0.89 (0.38–2.08)	0.783
no RT of prostate bed		17	12				
RT of prostate bed		85	37				
**RT Dose to the Prostate Bed (EQD2**_**α/β=1.5 Gy**_ **)**				0.51 (0.25–1.02)	0.058		
< 66 Gy		44	NR				
≥ 66 Gy		41	37				
**Concomitant ADT**				0.34 (0.16–0.72)	**0.005**	0.29 (0.13–0.65)	**0.003**
no ADT		71	27				
ADT		31	NR				
**Duration of ADT**				1.81 (0.45–7.27)	0.401		
≤ 12 months		19	NR				
> 12 months		11	NR				

## Discussion

In the present analysis we found similar oncologic results of WPRT and HPRT in PSMA-PET/CT- positive nodal recurrences after prostatectomy. There were no significant differences in BRFS, MFS and NRFS. After two years, BRFS, MFS and NRFS were 61%, 86%, 88% after WPRT and 57%, 90%, 82% after HPRT, respectively.

This analysis stands in line with previous retrospective datasets evaluating different radiotherapeutic approaches for nodal recurrent PC. Retrospective mono- and multi-institutional series analysing the outcome of SBRT of LNM reported progression-free survival (PFS) rates of approximately 30% after 2 years and distant PFS rates of approximately 40–50% after 2 years [4, 21–23]. In these series approximately 40 to 50% of patients received ADT and patients were mostly staged with choline PET/CT. Correspondingly, the prospective phase II trials ORIOLE and STOMP evaluating MDT in oligometastastic PC included also patients with LNM and reported PFS rates of 60% after 2 years [6, 7]. Patients in these trials did not receive ADT and were staged by conventional imaging or choline PET/CT. When looking at WPRT, there are also some retrospective studies reporting the outcome. Tamihardja et al. found a biochemical progression rate of 50.1% after 5 years. 71% of patients were staged by PSMA-PET/CT and 60% received ADT. PSMA-PET/CT was associated with a significantly better outcome compared with choline PET/CT [14]. Rogowski et al. found a BRFS of 72% after 2 years in a patient cohort completely staged by PSMA-PET/CT. 83% of patients received concomitant ADT [13]. The prospective OLIGOPELVIS GETUG P07 trial evaluated WPRT with SIB to LNM and 6 months of ADT and reported a BRFS of 58% after 2 years. However, all patients were staged by choline PET/CT [13]. Furthermore, there is a large retrospective multicenter analysis comparing SBRT and WPRT favoring WPRT with a MFS after 3 years of 68% (SBRT) and 77% (WPRT), respectively [16]. Patients were staged mostly by choline PET/CT and 23% (SBRT) vs. 60% (WPRT) of patients received ADT. Soldatov et al. performed a retrospective analysis evaluating PSMA-PET/CT-guided MDT including also patients with LNM recurrences and reported a biochemical progression in 43% of patients after 18 months [18]. Remarkably, in this series LNM were treated with an involved site approach (an external iliac LNM on the left side resulted in RT of the left-sided external iliac lymphatics). There is no clear information about concomitant ADT, but 24% of patients had ADT at time of PSMA-PET/CT.

All in all, compared to other publications we observed a comparably favorable oncologic outcome, probably due to PSMA-PET/CT staging and the large proportion of patients with concomitant ADT.

In our analysis, significant predictive factors in multivariate analysis were concomitant ADT, the application of a LNM boost and a dose to the pelvic lymphatics of > 50 Gy EQD2_α/β=1.5 Gy_. Concomitant ADT is a known predictive factor also described by other groups [13, 14]. Regarding the dose to the pelvic lymphatics there is insufficient data. The current NRG Oncology Group recommendations recommend 45–50.4 Gy [24]. Recent prospective trials including RT of the pelvic lymphatics used an EQD2_α/β=1.5 Gy_ of 42.4 Gy [25, 26], 46.0 Gy [27], 47.5 Gy [28] or 50 Gy [29]. This work indicates that choosing 50.4 Gy should be considered – at least in the setting of nodal recurrent patients - and that the optimal dose still needs to be found in the future. To our knowledge there is no data on the influence of a LNM boost on oncologic outcome, although this is often applied as in the prospective OLIGOPELVIS GETUG P07 trial using a SIB concept to LNM of 66 Gy in 30 fractions [15].

Of course, there are several limitations of this analysis. First, the retrospective and multi-institutional character of the dataset leads to heterogenous diagnostic and therapeutic procedures. For example, there were no standardized PET/CT protocols, no standardized recommendations for the use of ADT and no standardized contouring templates or RT dose prescriptions. Second, there were still differences in some of the patients’ and treatment characteristics (dose of LNM boost, PSA recurrence vs. PSA persistence, additional local recurrence) and the median follow-up. Even if univariate analysis showed that none of these parameters had a significant influence on the outcome in this dataset (see Table 2), PSA persistence in general is known to predict BRFS, MFS and OS [30, 31]. Furthermore, the combination of PSA persistence and LNM in PSMA-PET/CT can be regarded as synchronous metastatic disease having a worse prognosis than metachronous metastatic disease [32]. These factors limit the comparability of the WPRT and HPRT group as the WPRT group may have been at a higher risk for further progression.

Third, the dataset did not include some relevant information like the number of LNM or the pattern of LNM distribution (uni- vs. bilateral) which may have had an influence on the treatment decision of the treating physician. It is possible that the treating physicians opted for WPRT in the case of bilateral or multiple LNM, so that there could be a tendency to find more patients with high-risk factors in the WPRT group. Other relevant information was not complete in this dataset. For example, information regarding the pN status, the R status and the RT dose to LNM are missing in approx. 15% of the patients. Moreover, the duration of ADT, which is known to predict BRFS in locally advanced patients [33] or MFS in the salvage situation [34], is missing for more than two thirds of patients. Therefore, it is possible that the results of this study are biased by different ADT durations in both groups.

The intention of treating only the unilateral lymphatic pathways is to reduce the radiation volume and thus to reduce toxicity. Unfortunately, due to the retrospective and multi-institutional character of this analysis without standardized documentation of toxicity the dataset did not include this data. The SPPORT trial showed that adding WPRT to prostate bed RT + ADT increased the acute toxicity (CTCAE °2 or higher) from 37.7 to 44.6% but did not increase late toxicity [25]. The PEACE V-STORM trial did not observe any differences in acute toxicity (CTCAE °2 or higher) or quality of life (QoL) between MDT (sLND or SBRT) + ADT and MDT + ADT + WPRT [26]. In a retrospective multi-institutional comparison SBRT patients had less acute (3.0% vs. 0.3%) and late (1.0% vs. 10.5%) toxicity (°2 or higher) than elective nodal RT patients [16]. Nevertheless, with regard to toxicity there is no sufficient prospective data to compare SBRT and WPRT and, to our knowledge, no data to compare HPRT and WPRT with regard to their toxicity.

When interpreting the outcome of PSMA-PET/CT-guided RT also the limitations of PSMA-PET/CT have to be taken into account. Despite being the most advanced prostate cancer image modality the correlation of PSMA-PET/CT results with histopathological results revealed a sensitivity of only 40% in detecting LNM prior to pelvic lymph node dissection [35]. Thus, shrinking RT volumes has always the risk of missing more PSMA-PET/CT-occult LNM.

An interesting topic for future research is the metastasizing routes of LNM in PC patients. In the definitive setting there is evidence that 30–40% of nodal positive patients had LNM on the contralateral side of the dominant intraprostatic lesion [36] so that lymph node dissection or elective nodal RT is ideally performed bilaterally. In the postoperative setting with its changed lymphatic drainage there is no clear evidence about risk factors for bilateral lymphatic spread. After definitive prostate-only RT, failure pattern analyses showed that 55% of patients developing pelvic nodal recurrences had a common iliac involvement and 28% of patients had involvement of only one lymph node station. Risk factors for common iliac involvement were omission of ADT and a T3 stage [37]. All in all, defining subgroups of patients being appropriate candidates for a reduction of RT volumes like HPRT and also SBRT should be aimed. For this process it would be interesting to establish also risk factors for bilateral spread.

Furthermore, it will be also necessary to look beyond the local and radiotherapeutic treatment options. The EMBARK trial showed that a combination of enzalutamide and ADT in high-risk recurrent PC patients (PSA doubling time ≤ 9 months) led to a better MFS than ADT alone or enzalutamide alone. However, patients in this trial did not receive RT [38]. In a retrospective analysis of the STAMPEDE platform in primary and relapsed PC patients with LNM or other high-risk features adding abiraterone and prednisolone to standard of care improved MFS. However, in this analysis only 1–2% of the relapsed patients had LNM and pelvic RT was not part of the therapy [39]. Two phase II trials showed that adding enzalutamide to salvage RT of the prostate bed was safe and led to encouraging results in patients with high-risk biochemical recurrence. However, in these trials patients with (large) LNM were excluded and pelvic RT was not allowed [40, 41]. Altogether, further research is required to determine the role of advanced ADT and its combination with RT in patients with nodal recurrence.

## Conclusions

The present retrospective analysis compared the outcome of HPRT and WPRT in patients with PSMA-positive LNM recurrences after prostatectomy. Furthermore, it is the first report of HPRT results in PC patients. Despite using propensity score matching there were still imbalances between both groups and patients of the WPRT group may have been at a higher risk for further progression than patients in the HPRT group. All in all, no significantly different results were observed in both groups and results were favorable overall (2-yr BRFS approximately 60% in both groups). Concomitant ADT, RT dose to the pelvic lymphatics and application of a LNM boost were significant predictors of BRFS. Other radiotherapeutic approaches (SBRT or WPRT) will remain preferred treatment options due to better evidence. Nevertheless, this analysis shows that HPRT for selected favorable risk patients deserves further research including long-term follow-up, prospective trials and evaluation of toxicity and quality of life.

## Data Availability

The datasets generated analysed during the current study are available from the corresponding author on reasonable request.

## Abbreviations

ADT: Androgen deprivation therapy
BRFS: Biochemical recurrence-free survival
CI: Confidence interval
EQD2: Equivalent dose in 2 Gy fractions
HPRT: Hemi pelvis radiotherapy
HR: Hazard ratio
ISUP: International society of urological pathology
LNM: Lymph node metastasis
MDT: Metastasis directed therapy
MFS: Metastasis-free survival
NRFS: Nodal recurrence-free survival
NR: Not reached
PFS: Progression free-survival
PC: Prostate cancer
PSA: Prostate-specific antigen
PSMA-PET/CT: Prostate-specific membrane antigen-positron emission tomography/computed tomography
RT: Radiotherapy
sLND: Salvage lymph node dissection
SIB: Simultaneous integrated boost
SBRT: Stereotactic body radiotherapy
